# Polymerase chain reaction confirmed mycobacterium tuberculosis intermediate uveitis – analysis of 22 eyes of 14 cases from a tertiary care centre in South India: a retrospective study

**DOI:** 10.1186/s12348-022-00296-0

**Published:** 2022-07-11

**Authors:** Janani Sreenivasan, Anupreeti Jain, P. Neha Kamalini, M. K. Janani, Jyotirmay Biswas

**Affiliations:** 1 Department of Vitreo Retinal Services, Sankara Nethralaya, 18 College Road, Nungambakkam, Chennai, Tamil Nadu 600006 India; 2 Department of Uvea and Ocular Pathology, Sanakara Nethralaya, 18 College Road, Nungambakkam, Chennai, Tamil Nadu 600006 India; 3 Vision Research Foundation Referral Laboratory, Chennai, India

**Keywords:** Intermediate uveitis, Polymerase Chain Reaction, Mycobacterium tuberculosis, Real-time PCR, Nested PCR

## Abstract

**Background:**

To report the role of Polymerase Chain Reaction in confirming the diagnosis of presumed *Mycobacterium Tuberculosis (MTB)* Intermediate Uveitis.

**Method:**

Retrospective analysis of 22 eyes of 14 cases of presumed tubercular intermediate uveitis wherein intraocular fluid was tested for MTB DNA by Nested & Real-time PCR, based on clinical suspicion of tubercular aetiology. QuantiFERON TB gold test and High-Resolution CT Chest were done. Patients were treated with anti-tubercular therapy with oral steroids & immunomodulators. In the study, eleven were male (79%) and three female (21%). The median age was 34 years. Nested PCR for both IS 6110 & MPB 64 was positive in 64% of the cases, IS 6110 positive in 23% and MPB 64 positive in 15%. Real-time PCR was positive in 48% of the cases. Vision improved in 33% of cases, maintained in 57%, and worsened in 10% of cases.

**Conclusion:**

Presumed Tubercular intermediate uveitis can be confirmed by PCR of intraocular fluids. Anti-tubercular therapy with immunosuppression can improve vision and prevent recurrences in such cases.

## Background

Tuberculosis is a major health problem in India. It is the second leading cause of death by a single infectious agent worldwide [1]. Ocular TB is a recognized form of extrapulmonary TB associated with significant morbidity [2]. Ocular tuberculosis can manifest as anterior, intermediate, posterior or pan uveitis. It often poses a diagnostic dilemma due to its protean manifestations. If it is not treated adequately, it can lead to permanent loss of vision [1]. In recent years, the use of molecular biologic techniques based on DNA amplification to detect small amounts of genomic sequences from fluids or tissues has allowed us to diagnose or confirm infections that were previously difficult to detect [1]. Polymerase chain reaction (PCR) is one such technique for the evaluation of very small amounts of DNA or RNA by enzymatic amplification of nucleic acid sequences [3, 4]. The first use of PCR in detecting Mycobacterium tuberculosis (MTB) from the eye was reported from aqueous samples of two patients with active retinal vasculitis [3, 5]. Quantitative (qPCR) or real-time PCR is a variant of PCR that allows the quantification of nucleic acid in the sample and facilitates the monitoring of the progress of a PCR reaction in real-time [3, 6]. Although PCR positivity for MTB has been reported before in other tubercular uveitis entities (anterior, posterior) and retinal vasculitis, very few studies have reported PCR positivity for the same in intermediate uveitis [7–11]. We studied intraocular fluid by PCR in intermediate uveitis for mycobacterial tuberculosis DNA by both nested and real-time PCR. The objective of the study is to report the clinical profile, management and visual outcome of 14 cases of intermediate uveitis positive for Mycobacterium tuberculosis DNA by PCR of intraocular fluid (aqueous and vitreous). According to COTS-2 guidelines, either one or both immunologic tests (purified protein derivative PPD, interferon-gamma release assay IGRA) along with high resolution computed tomography (HRCT) of the chest suggestive of healed TB aetiology is required to start anti-tubercular treatment [12].

## Methodology

It was a retrospective case series wherein 22 eyes of 14 patients diagnosed with unilateral/bilateral intermediate uveitis with intraocular fluid were positive for Mycobacterium tuberculosis (MTB) DNA by PCR – Nested (both IS 6110 & MPB 64 gene) and/or real-time with minimum 1 year follow up were analysed.

The diagnosis of intermediate uveitis was made based on the Standardization of Uveitis Nomenclature (SUN) working group international workshop for reporting clinical data, which stated that it is a subset of uveitis where the vitreous is the major site of the inflammation, with or without peripheral vascular sheathing and macular oedema [13]. All patients had a detailed history including systemic illness recorded in the case records. The patients underwent complete ophthalmic examination including best-corrected visual acuity, intraocular pressure by applanation tonometer, slit-lamp biomicroscopy, and posterior segment examination with both slit-lamp biomicroscopy and indirect ophthalmoscopy at baseline and subsequent follow-up visits. Ancillary tests including fundus fluorescein angiography, optical coherence tomography with or without angiography, ultrasound biomicroscopy, etc., were done as and when indicated. Systemic assessment of all patients was done by an in-house internist. All patients underwent laboratory investigations like complete blood count (CBC), erythrocyte sedimentation rate (ESR), C-reactive protein (CRP), syphilitic screening by Venereal Disease Research Laboratory (VDRL), and treponema pallidum hemagglutination test, high resolution computed tomography (HRCT) chest, Mantoux test, tuberculosis (TB) interferon-gamma release assay (Quanti-FERON TB gold test) and serum angiotensin-converting enzyme (ACE) level were done. Also, magnetic resonance imaging (MRI) scan of the brain and immune markers consisting of rheumatoid factor (RF), anti-double-stranded deoxyribonucleic acid (anti-dsDNA), antineutrophil cytoplasmic antibodies (ANCA) were done whenever required. After ruling out other causes of intermediate uveitis, the decision to test MTB PCR in ocular fluids was taken if patients show positive results in one of the corroborative investigations suggesting tubercular aetiology which includes positive PPD test, IGRA assay, HRCT chest. PCR was done in the aqueous sample of 7 patients and the vitreous sample of 7 patients. The patients who were tested with vitreous samples had undergone diagnostic as well as therapeutic vitrectomy for non-resolving or recurring inflammation, despite maximal tolerated anti-inflammatory therapy.

DNA was extracted from the specimens (aqueous aspirate) using a Qiagen DNA extraction kit (Hilden, Germany) following the manufacturer's instructions. The extracted DNA was stored at -20 degrees C till further use. Nested PCR with 2 sets of primers targeting MPB 64 gene and IS6110 region were carried out [14]. Real-time PCR for the detection of the Mycobacterial load was estimated in the DNA extracts of the test sample using a commercial kit—Geno Sen’s® MTb Complex RG quantitative and the assay was performed on Rotor-Gene (Hilden, Germany) real-time PCR equipment based on Taqman principle.

All patients received treatment with oral steroids and four-drug anti-tubercular therapy. Those not responding to corticosteroids immunomodulators were added. The decision regarding treatment regimen in terms of initiation and duration of ATT and the addition of immunomodulatory therapy was taken in consensus with attending physicians in collaboration with respiratory disease physicians.

The primary outcome measure was changed in visual acuity, whether improved or maintained or deteriorated at the 1 year follow up. The secondary outcome measure was the proportion of patients with recurrence of inflammation after 6 months of initiation of treatment. The study was approved by The Institutional Review Board and adhered to the tenets of Helsinki.

## Results

**Fig. 1:**
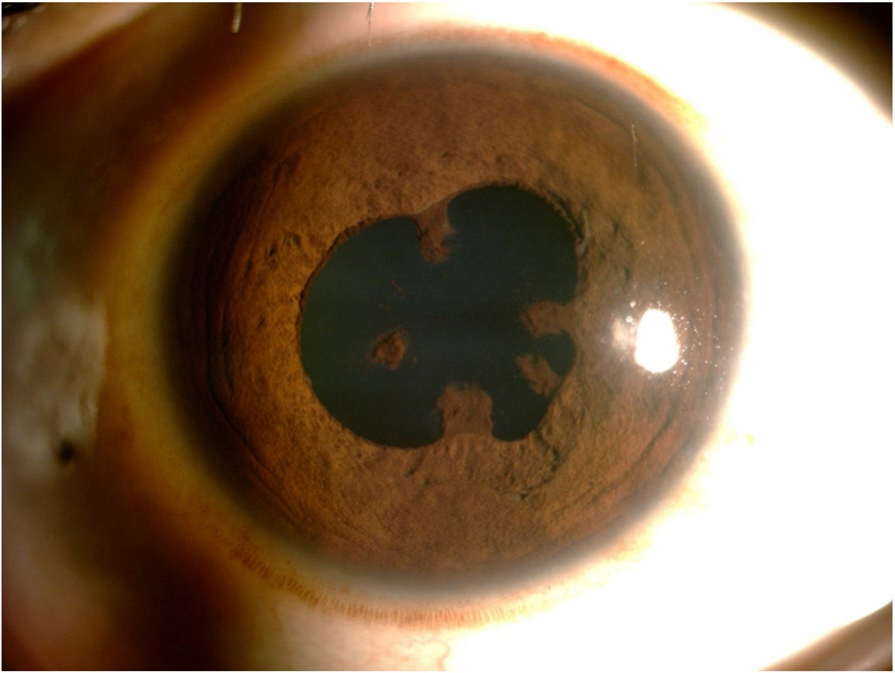
Slit-lamp photograph of right eye spill over anterior uveitis with broad-based posterior synechiae

**Fig. 2:**
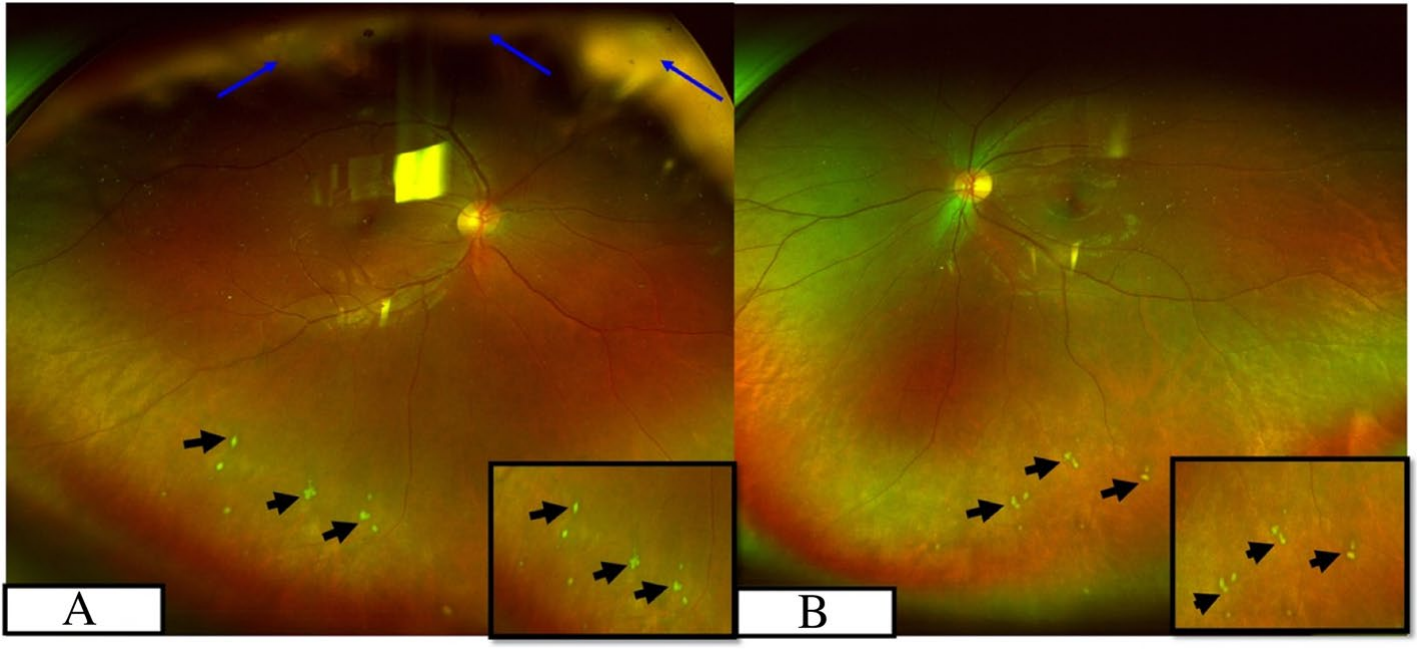
Wide-field fundus photo (pseudocolour fundus picture- Optos 200Tx imaging system, Optos PLC, Dunfermline, Scotland, UK) of the right eye (**A**) and left eye (**B**) of a patient whose aqueous sample was tested positive for PCR MTB. It showed relatively clear media with snowball vitreous exudates (black arrowheads) and attached retina. The right eye also had peripheral vitreous membranes (blue arrow)

**Fig. 3:**
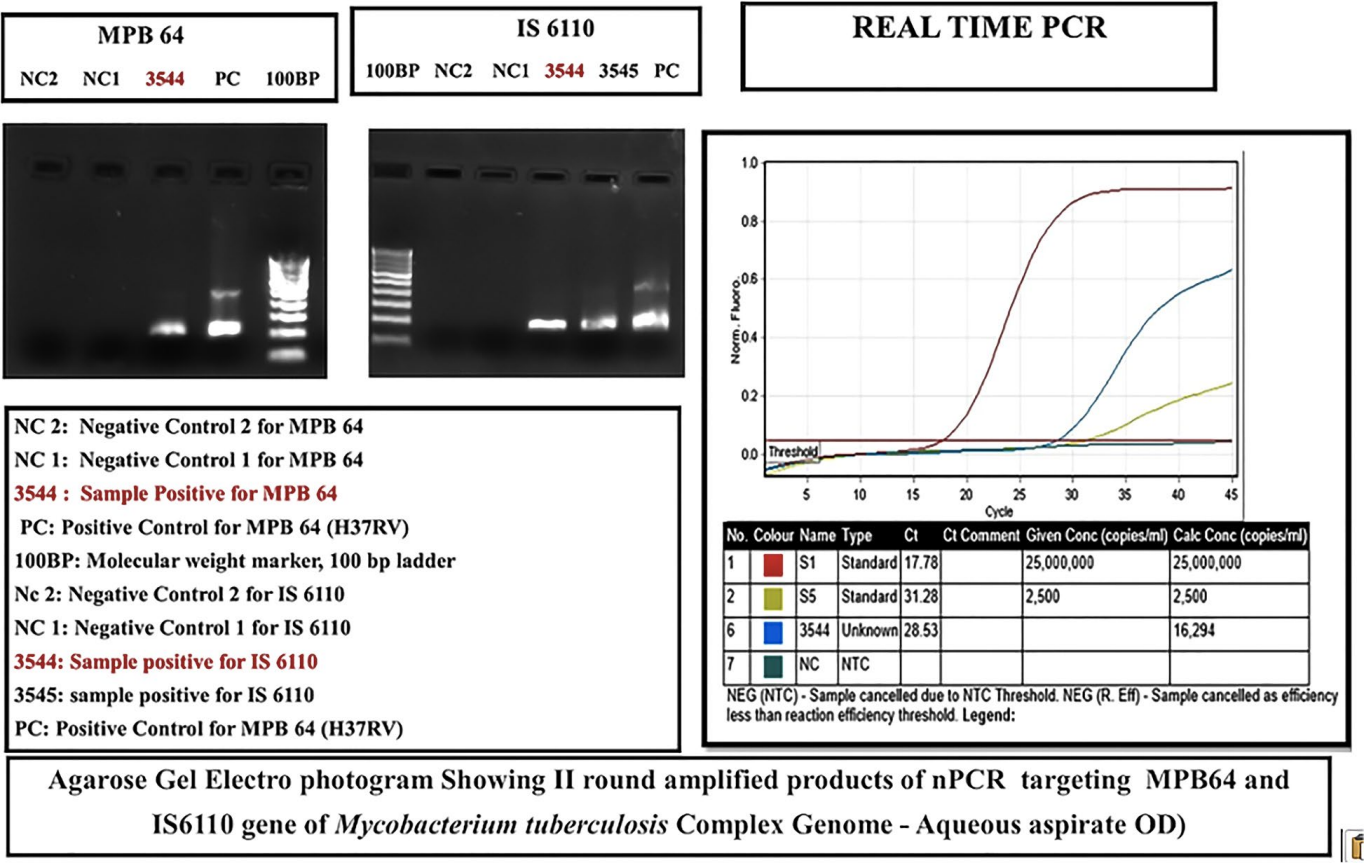
Aqueous sample of a patient which showed a positive result in Nested and Real-time PCR for MTB

**Fig. 4:**
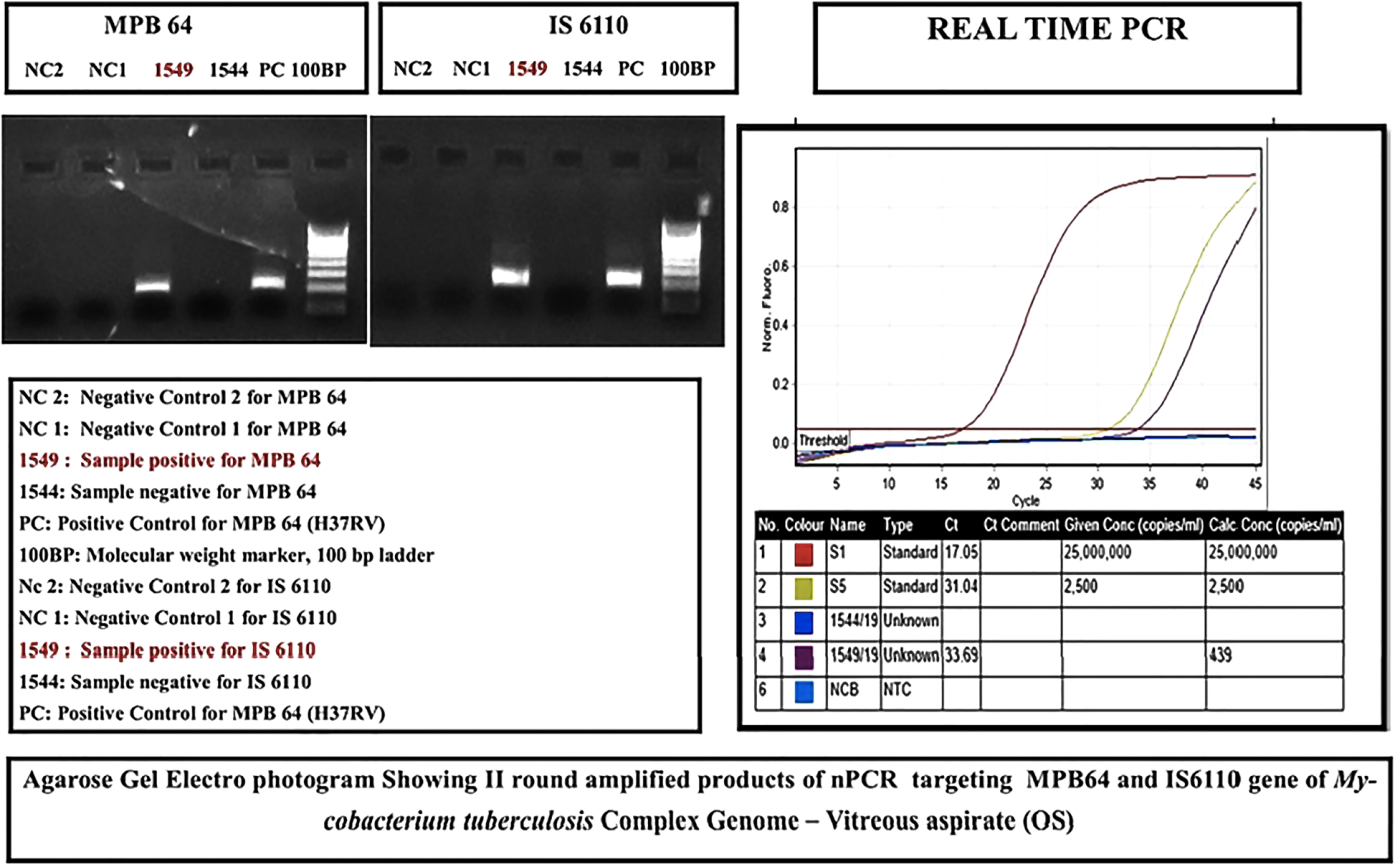
Vitreous sample of a patient which showed positive results in Nested and Real-time PCR for MTB

The study included fourteen patients, out of which 11 were males and 3 were females; The median age was 34 years (Range: 10-61 years). The disease was bilateral in 8 patients (57%). Defective vision (93%) was the most common presenting complaint, followed by floaters (70%). Twenty-three per cent of patients were previously treated with oral steroids and another 23% with posterior sub tenon steroid injections. The mean best-corrected visual acuity at presentation and at last follow up were 6/24 and 6/18 respectively. The anterior segment was quiet with broad-based posterior synechiae (Fig. 1) in twenty-nine per cent of patients. Vitreous was the most common finding, present in all the eyes and snowballs exudates were present in 33% of the eyes. Fundus examination (pseudocolour fundus picture- Optos 200Tx imaging system, Optos PLC, Dunfermline, Scotland, UK) of both eyes (Fig. 2A and B) of a patient whose aqueous sample was tested positive for PCR MTB showed relatively clear media with snowball vitreous exudates (black arrowheads) and attached retina and the right eye (Fig. 2A) also had peripheral vitreous membranes (blue arrow Fig. 2B). PPD was noted in 46%, positive HRCT in 38% and Quanti-FERON TB gold was positive in 46% of cases. All the three were positive in 1% of the patients. PCR was done in an aqueous sample of 7 patients and a vitreous sample of 7 patients. PCR results are shown in Table 1. Nested PCR for both IS 6110 & MPB 64 was positive in 64% of the cases. IS 6110 positive in 23% and MPB 64 positive in 15%. Real-time PCR was positive in 48% of the cases. Nested and Real-time PCR positivity in the aqueous sample of 7 patients (Fig. 3) and that of a vitreous sample of 7 patients (Fig. 4). All patients were treated with oral steroids with anti-tubercular treatment for 9 months after being evaluated by an attending in-house physician and a pulmonologist with initial 2 months of intensive treatment with all 4 drugs (isoniazid, rifampicin, ethambutol and pyrazinamide) followed by 7 months with 2 drugs (isoniazid and rifampicin). Posterior sub tenon steroid injections were given in 15% of the patients. Immunomodulator therapy was needed in 46% of the patients and the most common drug used was Mycophenolate Mofetil. Ocular complications encountered were epiretinal membranes (29% of eyes), cystoid macular oedema (14%), posterior subcapsular cataract (0.1%), hypotony (0.1%), choroidal neovascular membrane (CNVM) (0.1%). No systemic complications were encountered. Vision improved in 33% of the cases, maintained in 57% and worsened in 10% of cases. Factors associated with poor visual acuity include cystoid macular oedema, epiretinal membrane and cataract. Recurrences were observed in 23% of the eyes 6 months after initiation of treatment.

**Table 1:** PCR for MTB results of intraocular fluids of patients included in the stud

PCR	PERCENTAGE OF CASES POSITIVE
NESTED PCR (MPB 64 & IS 6110)	64%
PCR IS 6110	23%
PCR MPB 64	15%
REAL TIME PCR	48%

## Discussion

Mycobacterium tuberculosis is one of the important infectious agents responsible for intermediate uveitis. It was thought that eye shows hypersensitivity to the non-viable or tubercular bacilli DNA causing such uveitis. For a definite diagnosis of aetiology, isolation of the MTB bacilli from the vitreous sample, aqueous paracentesis or retinal biopsy is required.

Our study provided the clinical spectrum and visual outcome of patients with intermediate uveitis with positive PCR of intraocular fluids for MTB. The Pubmed search did not show any study on Real-Time/nested Quantitative PCR positivity for MTB in intermediate uveitis. PCR has emerged as a powerful tool for rapid detection of the mycobacterial genome, with high specificity and variable sensitivity. The utility of PCR analysis lies in important facts like ocular samples usually are tested negative for the acid-fast bacilli (AFB) with direct smear and culture due to the paucibacillary nature of the disease and the systemic involvement may not be present at the time of ocular disease, causing a delay in diagnosis and management [15].

PCR has a very low false-positive rate on intraocular fluids. External contamination leads to false-positive and polymorphism, specimen degradation, or failure to sample in the acute stages of disease-causing false-negative results [16]. Arora et al. evaluated the role of PCR for the detection of Mycobacterium tuberculosis in aqueous humour samples obtained from eyes with active uveitis and showed that it can be effectively used for the diagnosis of intraocular tuberculosis, which was supported by the review report by Gupta et al [17]. In our study, we performed PCR testing for presumed tubercular intermediate uveitis. As culture is difficult and time-consuming, presently, the use of real-time PCR can be done to establish tubercular aetiology, our study also proved PCR to be an important tool for rapid detection of the mycobacterial genome in suspected tubercular intermediate uveitis cases.

Parchand et al. discussed the clinical profile of intermediate uveitis in the Indian population and concluded that the addition of anti-tuberculosis therapy in cases of intermediate uveitis of presumed tubercular origin can reduce the recurrences [18]. According to them, patients were diagnosed with intermediate uveitis of presumed tubercular aetiology if there was a documented positive tuberculin skin test (10 mm of induration or more) at 48–72 h with evidence of vitritis, snowballs, or snow banking; and all known causes of infectious uveitis except TB and known non-infectious uveitis syndromes were ruled out. Madhavan et al. proved the presence of tuberculosis genome in the vitreous chamber fluid and the epiretinal membrane in Eales disease. They found that 5 out of 14 vitreous (20.8%) fluid samples were positive for the tuberculosis genome. In our study, we found that about 64% of the patients showed positivity for TB genome in nested PCR and 48% of cases showed positivity in real-time PCR in intermediate uveitis. The PCR results showcased circulating antigens of tuberculosis leading to an inflammatory response. In our study, fundus evaluation of patients with positive PCR (AC tap or Vitreous tap) demonstrated features of snowball opacities and vitritis. Our study provides the clinical profile of cases of intermediate uveitis with documented evidence of MTB DNA in ocular fluids. Of note is that 48% of patients in the study had actively multiplying copies of MTB DNA by Real-time PCR. In cases with signs and symptoms of intermediate tubercular uveitis but not fitting into COTS 2 criteria, ocular fluids PCR testing can also be considered as a diagnostic modality for appropriate diagnosis and treatment [19]. Figueira et al. described the role of anti-tuberculosis treatment in inflammatory diseases suggestive of tubercular aetiology [20]. The consensus was that the positively screened patients should be treated for active tuberculosis with 4 drugs (isoniazid, rifampicin, pyrazinamide and ethambutol) for 6–9 months. Patients should be reviewed at end of the initiation phase which is 2 months and the end of treatment, which is 9 months. Sixteen per cent of our patients had recurrences in one year. We considered MPB64 and IS6110 only for the PCR study based on the earlier reports, which suggest MPB64 is 10,000 times more sensitive than IS6110 for the diagnosis of tubercular uveitis [21]. In the Indian population, the lack or presence of relatively few copies of IS6110 has been recorded, which could lead to missing cases of probable intraocular tuberculosis. For the diagnosis of intraocular tuberculosis, the MPB64 gene-based PCR specific for MTB has been carefully studied and found to be more sensitive than the IS6110 PCR.

The study’s major limitations include small sample size, retrospective nature, lack of comparison with other causes of intermediate uveitis with intraocular fluids negative for PCR and relatively short follow up, lack of standardization of PCR, and not using multiplex PCR targeting more than one gene and low sensitivity. Further studies are needed to find out the role of PCR in intermediate uveitis of tubercular origin.

## Conclusion

Tuberculosis should be considered an important aetiology of intermediate uveitis in endemic countries like India. Intermediate uveitis of tubercular aetiology can be diagnosed by polymerase Chain Reaction of intraocular fluids. It can also be considered a diagnostic modality in cases with strong clinical suspicion of tubercular origin. Although anti-inflammatory therapy in the form of oral steroids with or without immunosuppression remains the mainstay of treatment of intermediate uveitis, the addition of anti-tubercular therapy to the treatment regime in PCR MTB positive cases can cause resolution of uveitis and reduce the recurrences.

## Data Availability

Available with the corresponding author.

## Abbreviations

PCR: Polymerase Chain Reaction
MTB: Mycobacterium Tuberculosis
HRCT: High-Resolution Coherence Tomography
qPCR: Quantitative Polymerase Chain Reaction
COTS: Collaborative Ocular Tuberculosis Study
IGRA: Interferon Gamma Release Assay
SUN: Standardization of Uveitis Nomenclature
PPD: Purified Protein Derivative
ATT: Anti Tubercular Treatment
